# Distal radial artery access is a safe and feasible technique in the anatomical snuffbox for visceral intervention

**DOI:** 10.1097/MD.0000000000033987

**Published:** 2023-06-16

**Authors:** Feng Jiang, Wen-Long Fan, Weiliang Zheng, Xia Wu, Hongjie Hu

**Affiliations:** a Department of Radiology, Sir Run Run Shaw Hospital, Zhejiang University School of Medicine, Hangzhou, Zhejiang, China.

**Keywords:** anatomical snuffbox, distal radial artery, transfemoral approach, vascular access, vascular intervention

## Abstract

Regarding the site of arterial access during the intervention, transracial intervention can reduce the risk of bleeding and vessel-related complications as well as improve patient comfort. Importantly, the distal radial artery (DRA) approach may reduce the incidence of radial artery occlusion and digital ischemia, but the feasibility and safety of DRA in performing subdiaphragmatic vascular interventions remain unclear. From January 2018 to December 2019, 106 patients were admitted to our department for visceral angiography and intervention by left distal radial artery accessing in an anatomical snuffbox. In total, 152 times of vascular interventions were performed during this period. Patients demographics, procedure details, technical success, and access site-related complications were recorded and evaluated. The mean age was 58.9 (range 22-86) years. Males accounted for 80.2%. Thirty-five patients (33%) had 2 or more procedures via the DRA approach. Technical success was achieved for 96.1% of procedures (146 cases) and 3.9% of cases failed to perform the intended procedure via the DRA approach (6 cases). The 4-Fr sheath was used in 86.8% of cases, and the 5 Fr sheath was used in the rest of the 13.2% of procedures. The rate of asymptomatic radial artery occlusion was 5.7% (6 of 106 patients). No patient suffered from distal limb ischemia after a long-time follow-up. Eight patients suffered postoperative local pain, transient numbness, or local bruised in the anatomical snuffbox without major complications. Patients with postoperative complications recovered quickly by using nonsteroidal anti-inflammatory drugs or without further treatment. Left distal radial artery access is safe and feasible as a new technique for visceral angiography and intervention.

## 1. Introduction

Concerning arterial access sites, the transfemoral approach (TFA) has been demonstrated to increase the rate of vessel-related complications and bleeding complications compared with the transradial approach (TRA).^[1–3]^ Femoral approach has given rise to increased mortality and hospital stay in percutaneous coronary intervention (PCI).^[4,5]^ Observational and randomized control trials comparing the TRA with TFA for coronary angiography and PCI showed a significant reduction in bleeding and access site complications when the TRA was used.^[5,6]^ The use of the TRA has several advantages including reduced bleeding risk, early ambulation, and improved patient comfort, no matter in PCI or peripheral vascular intervention.

The radial puncture is traditionally performed at 2 to 3 cm proximal to the radial styloid process of the hand.^[7]^ In 2017, a new site of radial artery puncture, distally to the traditional puncture site, in the anatomical snuffbox (AS) was described by Kiemeneij.^[8]^ The distinctive feature of the distal radial artery (DRA) segment is its location distally to the superficial palmar branch of the radial artery that joins the superficial palmar arch.^[9]^ Collateral vessels are communicating between the superficial palmar arch and deep palmar arche. Compared with the transitional radial artery access site, the DRA approach may reduce the rate of radial artery occlusion (RAO) and digital ischemia because of preserved blood flow in the superficial palmar arch.^[10]^ Moreover, the use of left DRA on the AS access and intervention can supply a comfortable position for both the patient and the operator.^[8]^ Recently, the distal radial approach is enthusiastically adopted by many operators for both left and right radial artery access.^[11,12]^ However, it remains unclear the feasibility of the use of DRA for vascular interventional below the diaphragm, and the safety and success rate of the use of the DRA approach compared with the use of TFA.

The purpose of this study was to show the feasibility and safety of DRA access in peripheral vascular interventions and to share the experience of the left DRA approach for peripheral vascular angiography and interventions.

## 2. Materials and methods

### 2.1. Patients

Patients who underwent noncoronary interventional radiology procedural between January 2018 and December 2019 in our institution were retrospectively reviewed. After approval was granted by the institutional review board in our hospital, electronic medical records were reviewed. Patient demographics and clinical data included age, sex, height, weight, platelet count, and international normalized ratio. Medical records were also reviewed to identify procedural details (disease, type of procedure, access side, sheath size, number of procedures, technical success), and post-procedural access-related complications reported during hospitalization or discharge follow-up visits.

### 2.2. Technical evaluative criteria

Technical success was defined as the successful completion of the intended procedure without any transverse to other access approaches or open surgery. It was deemed as a technical failure when there were attempts to cannulate any vessel previously designated as a primary target for therapeutic intervention or the crossover from distal radial to femoral approach. According to the quality improvement guidelines published by the Society of Interventional Radiology,^[13]^ complications were grouped as minor and major. Major complications included the need for prolonged hospitalization, unplanned increase in the level of care, permanent adverse sequelae, and death. Specific examples of major complications included blood transfusion, limb ischemia, pseudoaneurysm, and any access site complication requiring open surgical intervention. Minor complications included the need for additional nominal therapy and overnight admission for observation, loss of radial pulse without evidence of distal ischemia, blood loss not requiring transfusion, or hematoma without needing open surgical repair.^[14]^ Access site-related complications, such as hematoma, RA occlusion, and vascular perforation, were assessed. RAO was diagnosed using a combination of a physical examination at the clinic follow-up visit and a Doppler ultrasound examination. Perforation was identified by observation of extravasation on a radial arteriogram in an interventional procedure. Neurologic complications were documented, including transient ischemic attacks, reversible ischemic neurologic deficits, and stroke, which was defined as a new, persistent neurologic disability lasting more than 24 hours after the procedure.

Selection of the access site was based on operator preference and patient-specific characteristics noted at the time when each procedure was performed. For patients with hepatocellular carcinoma or liver-dominant metastasis, 1 or more times of transhepatic arterial interventions were needed. Before each time interventional procedure, the possibility of DRA access was tested using the Barbeau test and an ultrasound examination. The exclusion criteria included the radial artery diameter of <2 mm on ultrasound and the lack of a patent ulnar palmar arch in the left hand, as determined by the use of a Barbeau test. Patients displaying Barbeau waveform D, indicating inadequate ulnar palmar arch patency, were excluded from the DRA approach. In addition, patients with severe vascular tortuosity, radial artery occlusion, possessing a fistula for dialysis, or who were close to needing dialysis were also excluded.

### 2.3. DRA approach intervention procedure

**Figure 1.**
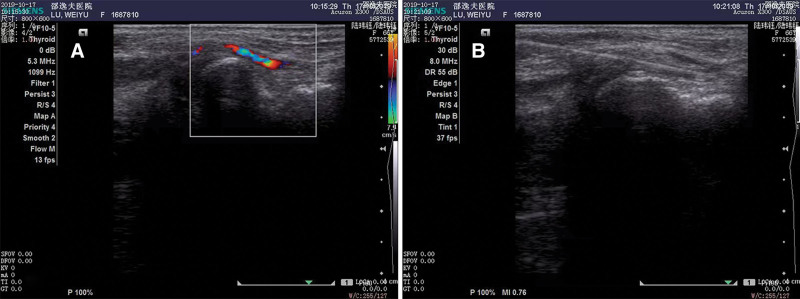
Ultrasound was used to help distal radial artery access. (A) The long-axis ultrasound images of the distal radial artery showed a color flow signal. (B) Under the guidance of ultrasound, the left DRA was successfully punctured with a 20-gage needle. DRA = distal radial artery.

**Figure 2.**
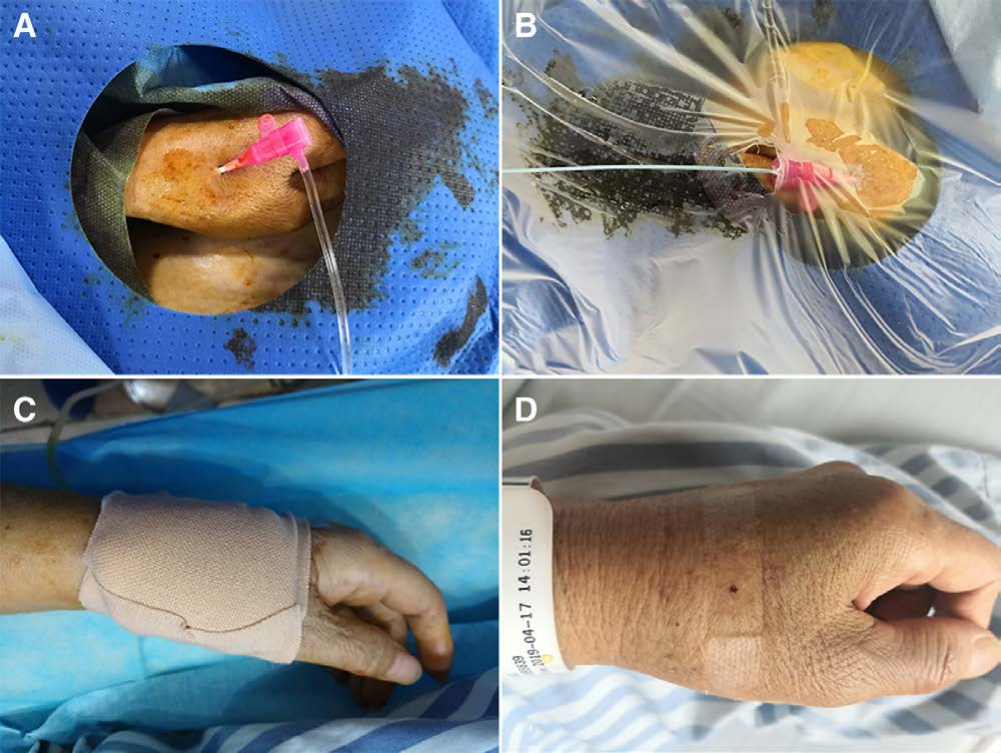
A 65-year-old male with HCC received transarterial chemoembolization. (A) The distal radial artery in the anatomical snuffbox was successfully accessed and inserted a 4 Fr hydrophilic sheath (Terumo). (B) A 4 Fr 125 cm MP A1 (I) catheter (Cordis) and a standard 0.035-inch × 180 cm access wire (Merit Medical) were used to traverse the subclavian artery. (C) After the interventional procedure was completed, compression was done using an expandable bandage preserving the blood flow of the ulnar artery. (D) No bruising or hematoma in the local area of the anatomical snuffbox was observed after removing the expandable bandage.

**Figure 3.**
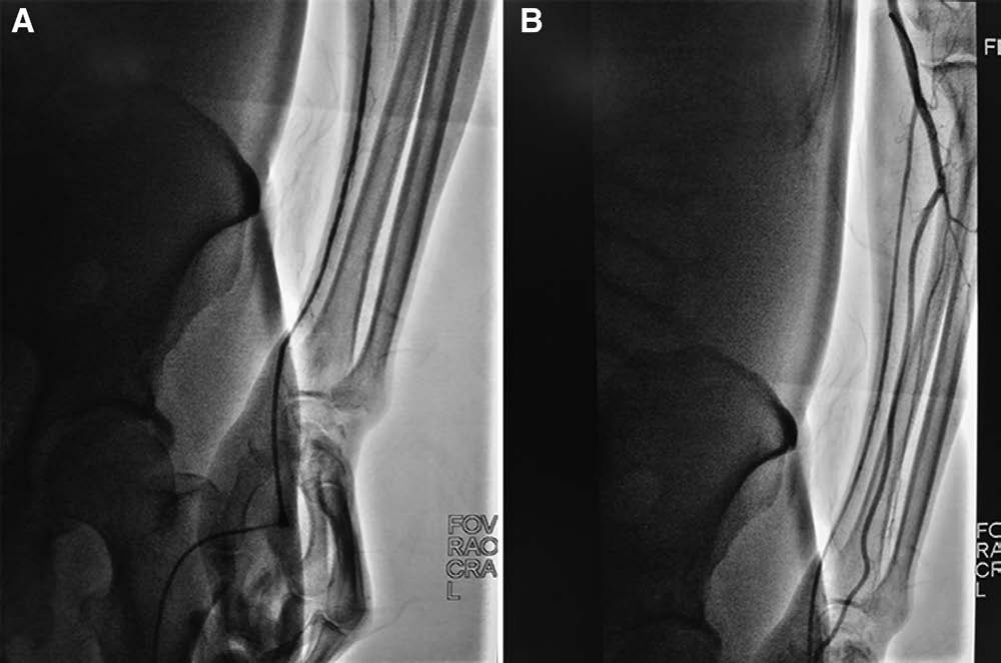
Radial artery angiography via the hydrophilic sheath. (A) Vasospasm was observed by sheath angiography after the hydrophilic sheath was successfully introduced. (B) The re-angiographic image showed vasospasm disappeared after a cocktail solution, containing 3000 U heparin, 200 mg nitroglycerin, and 2.5 mg verapamil, injected intraarterially via the hydrophilic sheath.

Informed consent was given before the procedure of DRA approach interventions. All procedures were performed with the help of the digital subtraction angiography suite Allura Xper FD20 (Philips Healthcare) or Innova 4100-IQ (GE Healthcare). Before the operation, the pulsation of the left DRA in the AS was reconfirmed by manual palpation. Disinfected the left hand with povidone-iodine solution and prepared for the access site. The left hand was positioned above the left groin with support underneath the left elbow and slightly abducted with the thumb underneath the other 4 fingers, thus making the fossa radialis more prominent. The operator was positioned on the right side of the patient preparing for a left distal radial artery puncture. The arterial puncture was attempted by palpation directly or with the help of ultrasound guidance at the discretion of the operator (Fig. 1). After local anesthetic with 2% lidocaine over AS, the DRA was punctured with a 20-gage needle. The successful puncture was followed by an insertion 0.035-inch straight guidewire. A 4/5 Fr hydrophilic sheath (Terumo) was placed over the wire utilizing the Seldinger technique in all cases (Fig. 2). To prevent vasospasm and reduce the risk of thrombosis, a solution including 3000 U heparin, 200 mg nitroglycerin, and 2.5 mg verapamil was injected intraarterially via the vascular sheath over 20 seconds (Fig. 3). Heparin was not administered or reduced in the case of an intervention to treat active hemorrhage. A 4/5 Fr 125 cm angiographic catheter was used to traverse the subclavian artery and engage the descending aorta over a standard 0.035-inch 180 cm guide wire. Under road-map imaging, navigation of the arm to the proximal axillary artery was required to prevent potential vascular lesions for artery loop and tortuosity. A microcatheter system was additionally used if required. After the interventional procedure, compression was done for at least 2 hours using an expandable bandage preserving the blood flow of the ulnar artery and permitting step-less regulation of compression. Repeat evaluation of the access site and radial pulse was performed for all patients before discharge and at the routine 1-month follow-up visit using Ultrasound and results were recorded.

### 2.4. Statistical analysis

All statistical analyses were performed using Statistical Package for Social Science 22.0 (IBM SPSS; IBM, Armonk, NY). Data are presented as mean ± standard deviation or median (interquartile ranges) for continuous variables and proportions for categorical variables.

## 3. Results

### 3.1. Characteristics of the patients

From January 2018 to December 2019, a total of 106 patients undergoing DRA approach interventional procedures at our institution were recorded. Their mean age was 58.9 (range 22–86) years. Male patients (n = 85) accounted for 80.2%. Thirty-five patients (33%) had 2 or more procedures via the DRA approach. In the sum of 152 DRA approach interventional procedures was performed involving the repeat procedure cases. For patients who received repeated procedures, the mean time interval between repeat DRA access was 2.3 months (range, 1 to 13 months). In 1 patient with hepatocellular carcinoma, 4 times repeated transarterial chemoembolization procedures were successfully performed via the DRA approach without crossover or access site-related complications (Table 1). Etiologic diseases of patients undergoing the DRA approach procedure were including, 81.1% of liver malignant tumors, 4.7% of gastrointestinal tumors, 5.7% of hepatic hemangioma, 1.9% of acute postoperative renal bleeding for percutaneous nephrolithotomy, 4.7% of liver cirrhosis and splenomegaly, and 1.9% of patients with a uterine fibroid tumor. The interventional procedures performed in our department included 126 cases of transarterial chemoembolization, 12 cases of partial spleen artery embolization for hypersplenism, 11 cases of transarterial angiography and embolization of gastrointestinal tumor or hepatic hemangioma, and 3 cases of uterine artery embolization.

**Table 1:** Patients’ demographic characteristics.

Characteristic	Values
Number of patients	106
Age in yr (mean, range)	58.9 (22–86)
Sex	
Male	85 (80.2%)
Female	21 (19.8%)
Underlying diseases	
Liver cancer	86 (81.1%)
Gastrointestinal tumor	5 (4.7%)
Hepatic hemangioma	6 (5.7%)
Bleeding	2 (1.9%)
Hypersplenism	5 (4.7%)
Uterine fibroid tumor	2 (1.9%)
Number of DRA procedures	
1	71 (67.0%)
2	25 (23.6%)
3	9 (8.5%)
4	1 (0.9%)
Cases of DRA intervention	152
Weight (mean, range)	63.8 (45–124)
Height (mean, range)	168.2 (152–185)
Coagulation	
INR (mean, range)	1.1 (0.9–1.9)
Platelet count × 10^9^/L (mean, range)	137.0 (34–433)
Procedures	
TACE	126 (82.9%)
TAE	11 (7.2%)
PSE	12 (7.9%)
UAE	3 (2.0%)

DRA = distal radial artery, INR = international normalized ratio, TACE = transarterial chemoembolization, TAE = transarterial angiography and embolization. PSE = the partial spleen artery embolization, UAE = uterine artery embolization.

### 3.2. Study outcomes

For 152 cases of the DRA approach procedure, technical success was achieved for 96.1% of procedures (146 cases) and 3.9% of cases failed to perform the intended procedure via the DRA approach (6 cases) (Table 2). In 1 case, after successful DRA access, a severe radial artery spasm occurred and the vasodilator could not reverse the vasospasm. This made advancing the catheters impossible, and TFA was performed to continue the procedure with the patient’s anxiety increased. In 2 cases, the radiologist failed to try to cannulate to the descending aorta for the acute angle between the aortic arch and the left subclavian, although Cobra 2-shaped catheter and/or Simmons II catheter were used. In the other 3 cases, after successful DRA access, advancement of the guidewire in a patent radial artery failed, and the procedure was successfully performed crossing over to TFA. No conversions to TFA resulted from inadequate device length or an inability to cannulate the access artery of interest. Most of these procedures (132 cases, 86.8%) were performed with the use of a 4 Fr sheath and 4 Fr angiographic catheter. Five-Fr sheaths and angiographic catheters were used in the rest of 13.2% (20 cases) procedures including these 6 cases crossing over to TFA. The mean time to puncture of DRA was 2.4 minutes, and ultrasound guidance was used in 42.1% of cases. The mean number of DRA access attempts per patient was 1.3 (range, 1 to 5). The mean total fluoroscopy time was assessed as 13.5 minutes (range, 2.6 to 45.2 minutes). The total complication rate of the DRA approach was 6.6% (10 of 152 cases). Five cases of postoperative local pain of the ipsilateral hand or numbness were recorded during hospitalization. Symptoms quickly disappeared by treatment with the use of nonsteroidal anti-inflammatory drugs alone in all cases. Three cases of small ecchymosis or hematoma at the puncture site were reported after the procedure. The complications spontaneously recovered without additional treatment. For patients who underwent 1 or more times DRA procedures, the rate of asymptomatic RAO was 5.7% (6 of 106 patients) and no patient suffered from distal limb ischemia after a long-time follow-up. For the RAO patients requiring another interventional procedure, TFA was the first choice to perform. No major complication was found in any of the DRA patients. No neurologic complications or contrast medium-induced nephropathy were recorded in our study.

**Table 2:** Procedure characteristics of patients undergoing left distal radial approach angiography and interventions.

Characteristics	Results
Cases	152 (100%)
Technical success	146 (96.1%)
Technical failure	6 (3.9%)
RA spasm	1
Acute aortic arch	2
Failed insertion of sheath	3
Cross to TFA	6 (3.9%)
Sheath size	
4-Fr	132 (86.8%)
5-Fr	20 (13.2%)
Access time (mean, range) (min)	3.4 (2.2–8.6)
Average attempts (mean, range)	1.3 (1–5)
Ultrasound guidance	
Yes	64 (42.1%)
No	88 (57.9%)
Fluoroscopy time (mean, range) (min)	13.5 (2.6–45.2)
Complications	10 (6.6%)
Local pain/Numbness	5
Local bruised/hematoma	3
Asymptomatic RAO	6
Major	0
Patient RAO rate	6 (5.7%)

RA = radial artery, TFA = transfemoral approach, RAO = radial artery occlusion.

## 4. Discussion

Owing to the lower risk of access complications and better patient comfort compared with femoral access, proximal radial access is the most commonly used access site and was given a Class I recommendation in the 2018 European Society of Cardiology coronary revascularization guidelines.^[15]^ In a recent issue, Aoi et al^[12]^ compared the clinical outcomes of distal and proximal radial access cases for cardiac catheterization. DRA was obtained in about 99% of patients, and the crossover rate was no more than 2%. There was no difference in the success rate of radial access or crossover rate from radial to femoral access between the 2 groups. In our study, the successful DRA was obtained in 96.1% of cases with a 3.9% crossover to femoral access. Although the access success rate is similar, the distal radial artery is more challenging to access, requiring a longer time thus making it less appealing for patients and radiologists. Compared with 7.3 minutes of mean DRA access time in Aoi report, the mean access time in our study was 3.4 minutes. However, the definition of access time in previous reports was not quite the same. The access time in our study was defined as the time from lidocaine injection to sheath introduction into the DRA.

In our experience, some factors may prolong the access time including the small diameter of DRA, variations in the palmar arches, and the unproficiency of the radiologist. Sometimes, we need a few times trying to puncture the artery, even if there is a bounding pulse in the AS area. Typically, proximal RA access is easy to perform without extra Ultrasound guidance in most patients. The smaller diameter of DRA made it more challenging to access than proximal RA. In prior reports, the diameter for the distal radial artery was approximately 2.4 mm compared with 3.1 mm of the conventional radial artery site, which may not be insignificant with a difference of nearly 20%.^[16]^ In our study, Ultrasound usage accounted for 42.1% of cases similar to Aoi’s reported 34.2% of Ultrasound used. Also, the small diameter of DRA may limit the size of sheaths that can be inserted. Kim et al^[17]^ showed that the average diameter of the distal radial artery was 2.57 mm, and a 6-Fr sheath was successfully performed in all 117 patients in their study. In Mazzarotto et al^[11]^ report, even 7 Fr Glidesheath was used to introduce the DRA in local AS for coronary chronic total occlusion interventions. In our study, 4 Fr sheath was used in 86.8% of cases, and 5 Fr sheath was used in the rest 13.2% of cases. No introducer sheath with a diameter larger than 5 Fr was used, and the radial sheath and catheter selection were appropriate with the left distal radial artery in AS. In addition, no resistance was reported when advancing the guidewire and catheters through the introduced sheath in AS, meaning that the angle of the introduced sheath was coaxial with the forearm left radial artery.

The right radial artery is the most commonly accessed site for most patients receiving PCI, as it is more convenient for the patient and the operator than the left radial artery.^[18]^ However, it is convenient for the operator to puncture the left hand and manipulate the angiographic catheter on the right side of the patient. And patient’s left arm was positioned over his/her abdomen toward the right groin without needing any support. Hence, advancing the catheters and anchoring at the aortic arch is easier from the left radial artery than from the right radial artery.^[19]^ Also, for vascular interventional below the diaphragm, it may shortage of the distance from the access site to the intended vessel through left RA access.^[20]^ Moreover, most of the patients are right handed. Through left RA access, no restricting was needed for the right hand after the procedure, especially for people with professions requiring right-hand dexterity.^[9]^

In previous reports, complete hemostasis was achieved in almost 3 to 6 hours with a slight compression bandage with an air sac over the access site for the proximal RA access site.^[20–22]^ In our study, complete hemostasis was achieved with a slight compression bandage with gauze applied over AS. And DRA in the AS indeed had significantly reduced hemostasis time: approximately 2 hours for cases without coagulation function abnormal. However, longer hemostasis time was inevitably needed for cases with increased risks of bleeding including obvious thrombocytopenia, application of anticoagulation, and higher dose of heparin usage. Post-operational RAO is a common complication, which is typically asymptomatic without needing further medical intervention. And it is reported to be 1% to 10% in patients undergoing proximal TRA intervention.^[23–25]^ As per the sane with the previous report, the rate of RAO in our study was 5.7%, which included patients who received 1 more time DRA approach interventional procedure. In a previous randomized comparison study, Pancholy et al^[26]^ demonstrated that patent hemostasis of the radial artery significantly decreased the rate of RAO. Furthermore, a shorter duration of hemostatic compression for 2 hours compared to 6 hours was associated with a lower incidence of RAO.^[27]^ In our experience, accessing this DRA distal to the bifurcation may translate to the advantage of shorter hemostasis time, which may contribute to the low rate of RAO. Moreover, smaller hydrophilic sheath utility, without a diameter larger than 5 Fr, may also accelerate the recovery of radial artery wounds. Thus, reducing the occurrence of RAO.

Other access site-related complications included postoperative local pain, transient numbness, and local bruised in the AS. All of the symptoms were self-recovered or recovered by nonsteroidal anti-inflammatory drugs without further treatment. No major complication was reviewed in our study. To some extent, the DRA approach was as safe as TRA interventional procedure. As same with proximal RA access, certain patient populations may derive particular benefits from the use of the TRA, including obese patients, those with a bleeding diathesis, and elderly patients.^[28]^ Particular anatomic limitations may preclude the use of the TFA or may make the TRA a better option for use in some patients.

The mean fluoroscopy time recorded was 13.5 minutes. Due to a different kind of operation, and different operator variations in fluoroscopic, and DSA protocol, the dose area product was not documented. Loewenstern et al^[29]^ showed no significant difference in patient radiation dose or fluoroscopy time between transracial and transfemoral access of transarterial radioembolization patients, similar to the results recorded in patients undergoing percutaneous coronary intervention or patients treated with visceral embolization.^[30,31]^

Some limitations of our study were listed as follows. First, the retrospective nature of our study is subject to selection bias. Second, access site selection was based on operator preference and patient-specific characteristics at the time of each procedure, which contributes to another selection bias and may result in an underestimation of the true success rate. Finally, the observed results may not be generalizable to all institutions. All in all, DRA access in the anatomical snuffbox is a safe and feasible technique for peripheral vascular angiography and intervention.

## 5. Conclusion

For patients undergoing peripheral vascular intervention appear to be well-suited for the DRA approach. The present study shows that the DRA in the AS is a safe and feasible access option for visceral intervention associated with a low rate of complication. The advantage of this new technique warrants further exploration.

## Author contributions

**Conceptualization:** Hongjie Hu.

**Data curation:** Feng Jiang, Wen-Long Fan, Weiliang Zheng.

**Formal analysis:** Wen-Long Fan, Weiliang Zheng.

**Investigation:** Feng Jiang, Wen-Long Fan, Xia Wu.

**Methodology:** Feng Jiang, Wen-Long Fan, Weiliang Zheng.

**Software:** Wen-Long Fan, Xia Wu.

**Writing – original draft:** Feng Jiang.

**Writing – review & editing:** Hongjie Hu.
